# Supplementary material for: Exposure to Ionizing Radiation Triggers Prolonged Changes in Circular RNA Abundance in the Embryonic Mouse Brain and Primary Neurons
Source: Cells. 2019 Jul 26;8(8):778. doi: 10.3390/cells8080778 (PMC6721538; doi:10.3390/cells8080778)
Supplement: Supplementary file 1 [file cells-08-00778-s001.pdf]

Table S1. Primers used for RT-qPCR.

Transcript	Forward primer (5′-3′)	Reverse primer (5′-3′)
Gapdh	AAGGTCATCCCAGAGCTGAA	CTGCTTCACCACCTTCTTGA
Tbp	CCAGAACAACAGCCTTCCAC	GTGGAGTAAGTCCTGTGCCG
Myc	TGCTGCATGAGGAGACACC	AGGGGTTTGCCTCTTCTCC
circ-0000604	GTTTGTTGTCATCTCTCGGGCTAC	CCACAGGGTTCAGGAAGTCTC
circ-0005721	GTTTGTTGTCATCTCTCGGGCTAC	TGAGTATGAGAAGGGCTCAGAGG
circ-0005724	GGTGCCTAGAAATCCTGATAAGAGT	CCGGCATGAAGACTTCCATC
Lin_Pvt1	GTCTGTGATGTCTTCAGCAATGG	CGACAATTCAGTCAAGGATCCAG
circ-0009172	GGTCCCAAGTGTAAGTCAGAATG	TCTGATTACATCCCACGTTTTGTC
circ-0009174	GCTGATGCTTGTACCACATTCC	AGTCACAATGAACGAAAGCCAAG
circ-0009175	GTCCAGTATAGCAGTCCGACTC	AGTCACAATGAACGAAAGCCAAG
circ-0009176	AAGCCTTCTGCTCTCAGGTTC	AGTCACAATGAACGAAAGCCAAG
Lin_Ano3	AAGGACTCTACCCTTAAGTGTTCC	GGAGACGAGATCGATCATAGTTGG
circ-0006330	GGCTGGCACTACCAGGATATAG	CCTTTGTGGGTCTCCTGTAACC
Lin_Sec14l5	CAGCTTCTGTGCTCTCTCTG	CCATGACCAGCTCGAATGG
circ-0001605	CCAGATGATGCAGACCCATCG	CTCAGGCATCCATACTCCTCAC
circ-0013703	ACATGGCAATGGCAAAACTTCC	CTCAGGCATCCATACTCCTCAC
circ-0013704	CGTTCCTGGCAGGCTGAG	GGAATCCTCCTGCAGTAGGTTG
Lin_Rnf169	CTAGACTCTGAGGTCCCTCAC	GTCTGTGCCTCAAACGCAG
Qki5	CTGTCATGCCAAACGGAAC	GATGGACACGCATATCGTG
Qki6	CTGTCATGCCAAACGGAAC	CGTTGGGAAAGCCATACCT
Qki7	CTGTCATGCCAAACGGAAC	GACTGGCATTTCAATCCACTC
